# Fine‐scale population structure of the northern hard clam (*Mercenaria mercenaria*) revealed by genome‐wide SNP markers

**DOI:** 10.1111/eva.13577

**Published:** 2023-07-10

**Authors:** Ann J. Ropp, Kimberly S. Reece, Richard A. Snyder, Jingwei Song, Ellen E. Biesack, Jan R. McDowell

**Affiliations:** ^1^ Virginia Institute of Marine Science, William & Mary Gloucester Point Virginia USA

**Keywords:** DArTseq™, genotyping by sequencing, isolation by distance, *Mercenaria mercenaria*, northern hard clam, population structure

## Abstract

Aquaculture is growing rapidly worldwide, and sustainability is dependent on an understanding of current genetic variation and levels of connectivity among populations. Genetic data are essential to mitigate the genetic and ecological impacts of aquaculture on wild populations and guard against unintended human‐induced loss of intraspecific diversity in aquacultured lines. Impacts of disregarding genetics can include loss of diversity within and between populations and disruption of local adaptation patterns, which can lead to a decrease in fitness. The northern hard clam, *Mercenaria mercenaria* (Linnaeus, 1758), is an economically valuable aquaculture species along the North American Atlantic and Gulf coasts. Hard clams have a pelagic larval phase that allows for dispersal, but the level of genetic connectivity among geographic areas is not well understood. To better inform the establishment of site‐appropriate aquaculture brood stocks, this study used DArTseq™ genotyping by sequencing to characterize the genetic stock structure of wild clams sampled along the east coast of North America and document genetic diversity within populations. Samples were collected from 15 locations from Prince Edward Island, Canada, to South Carolina, USA. Stringent data filtering resulted in 4960 single nucleotide polymorphisms from 448 individuals. Five genetic breaks separating six genetically distinct populations were identified: Canada, Maine, Massachusetts, Mid‐Atlantic, Chesapeake Bay, and the Carolinas (*F*_ST_ 0.003–0.046; *p* < 0.0001). This is the first study to assess population genetic structure of this economically important hard clam along a large portion of its native range with high‐resolution genomic markers, enabling identification of previously unrecognized population structure. Results of this study not only broaden insight into the factors shaping the current distribution of *M. mercenaria* but also reveal the genetic population dynamics of a species with a long pelagic larval dispersal period along the North American Atlantic and Gulf coasts.

## 1 INTRODUCTION

Aquaculture is growing rapidly worldwide, and sustainability is dependent on understanding current genetic variation and levels of connectivity among populations. Genetic data are essential to mitigate the genetic and ecological impacts of aquaculture on wild populations and guard against unintended human‐induced loss of intraspecific diversity in aquacultured lines. When wild and captive animals interbreed, the impacts on the wild populations can include loss of genetic diversity within and between populations and disruption of patterns of local adaptation via outbreeding depression leading to a decrease in fitness (Glover et al., 2017; Laikre et al., 2010; Sylvester et al., 2019; Waples et al., 2016). For aquacultured animals, an understanding of the wild population structure and levels of genetic diversity is critical to selecting animals with desired traits and supplementing brood stocks for efficient production and preventing inbreeding depression from unintended loss of genetic diversity in the domestication process (Li et al., 2017; Mather & de Bruyn, 2003; Miller et al., 2006; Purcell et al., 2015). Given these needs for genetic information and the decreased cost of next‐generation sequencing, it is “inexcusable not to know” the genetic makeup of lines and families used for aquaculture and restoration whenever possible to mitigate adverse effects (Ryman et al., 1995).

Bivalve mollusks have life history strategies that are unique compared to other marine species, making molecular studies of their population structure more challenging. These traits include a high inbreeding load, segregation distortion (Plough, 2016), and higher levels of polymorphism than most other animals (Curole & Hedgecock, 2005; Hedgecock et al., 2005; Reece et al., 2004). Single nucleotide polymorphisms (SNPs) are estimated to occur every 30–50 base pairs throughout the genomes of mollusks (Curole & Hedgecock, 2005; Garvin et al., 2010; Hedgecock et al., 2005; Reece et al., 2004) compared to one every 116 bp in channel catfish (Sun et al., 2014) and one every 137 bp in the European hake (Milano et al., 2011). These high levels of polymorphism have made using more traditional primer‐targeted markers like microsatellites challenging for genetic studies of mollusks due to the high incidence of null alleles caused by variation in primer‐binding sites. Advancements in high‐throughput genotyping by sequencing (GBS) and bioinformatics have enabled the rapid identification of polymorphic loci without reliance on specific sets of primers, making it possible to identify thousands of SNPs that can be screened to develop genetic resources for aquacultured bivalve species (Davey et al., 2011; Elshire et al., 2011).

The northern hard clam or northern quahog, *Mercenaria mercenaria* (Linnaeus, 1758), hereafter hard clam, is a marine bivalve inhabiting soft substrates of mud, sand, and shell fragments in coastal lagoons and estuaries from the Gulf of St. Lawrence, Canada, through the northern Gulf of Mexico (Mackenzie et al., 2002). The hard clam has been the target of a wild harvest fishery along portions of its native range and has become an economically important aquaculture species in the Mid‐Atlantic and southeastern USA (Whetstone et al., 2005). Globally, the value of aquacultured hard clam production was estimated to be $78 million in 2017 (FAO, 2018). Virginia is the largest producer of aquacultured hard clams in the USA, with farm sales estimated at $58 million in 2021 (Virginia Marine Resources Commission fisheries statistics), making it the most economically valuable aquaculture species in the US Mid‐Atlantic region.

The life history of the hard clam is similar to that of other marine bivalves, having a pelagic larval stage, a benthic adult stage, high fecundity, and a relatively short time to maturity (Carriker, 2001; Eversole, 2001), characteristics that can shape genetic population connectivity. Hard clams can grow to a maximum shell length (anterior–posterior parallel to the hinge) of about 120 mm and, using sclerochronological analysis, have been aged up to 106 years (Ridgway et al., 2011; Roegner & Mann, 1990). They are protandrous consecutive hermaphrodites with separate sexes and no sexual dimorphism, establishing their definitive sex by either transitioning to female or remaining male (Eversole, 2001; Loosanoff, 1936; Roegner & Mann, 1990). Hard clams have high fecundity and broadcast spawn from spring through fall depending on latitude, with timing and the length of the spawning season affected by temperature and food availability (Eversole, 2001; Roegner & Mann, 1990). The pelagic larval duration (PLD) is temperature dependent, lasting 18–24 days in 18°C water and 7–14 days in 30°C water, until the clams reach the proper size and development for metamorphosis (Mackenzie et al., 2002). Juveniles and adults are considered to have a sedentary life in the benthos due to their limited post‐metamorphosis mobility, so the interactions among PLD, hydrodynamics, geography, and other physical factors are thought to shape genetic connectivity (Cowen & Sponaugle, 2009).

The population genetics of wild hard clams have been investigated in two previous molecular studies. Dillon and Manzi (1992) examined isozymes in clams at the northern limit of their range and resolved three regional populations: (1) Prince Edward Island (PI), Canada, (2) Maine, and (3) Massachusetts, USA. Baker et al. (2008) used the mitochondrial cytochrome oxidase subunit I (COI) gene to examine population structure from PI, Canada, to Cedar Key, Florida, with a sampling focus within Florida. They found three genetically distinct populations separated by a strong genetic break at Cape Hatteras, North Carolina, and a weaker genetic break at Cape Cod, Massachusetts (Baker et al., 2008). Although isozymes and allozymes are well suited for population studies and the mitochondrial COI gene is useful for understanding the phylogeographic history of a species (Allendorf, 2017), both types of markers have limited capabilities for fine‐scale resolution of population structure.

The objective of this study was to determine the population structure and genetic diversity of wild hard clams along a large portion of their native range using thousands of SNPs identified through genotyping by sequencing (GBS). The fine‐scale population structure delineated by this study allows a better understanding of the mechanisms involved in maintaining population structure for marine bivalves along the North American east coast. Additionally, it provides baseline measures of standing genetic variation in wild populations that may be valuable for monitoring and quantifying changes in genetic diversity and effective population size in the face of warming coastal waters and proliferation of genetically focused aquaculture stocks.

## 2 MATERIALS AND METHODS

### 2.1 Sample collection and DNA isolation

**FIGURE 1:**
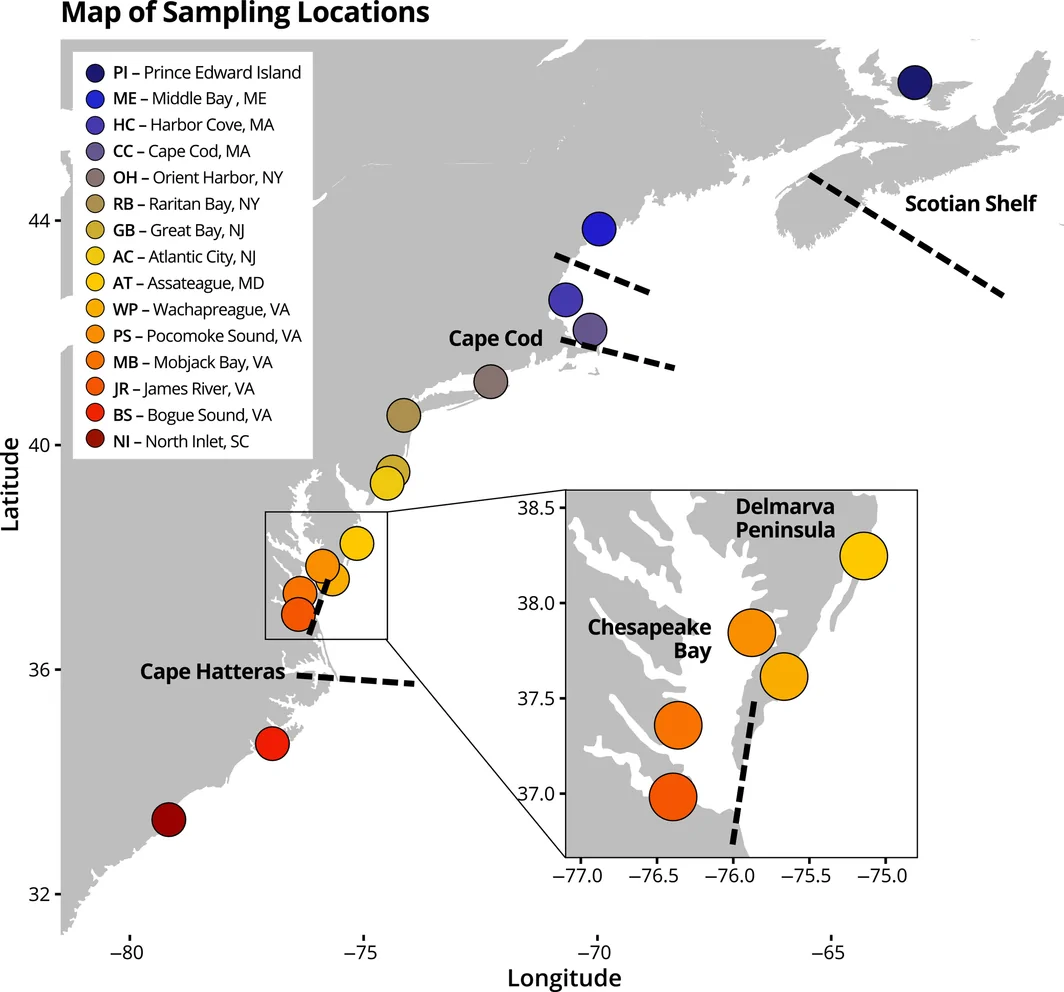
Map of sampling locations: Prince Edward Island, Canada (PI), Middle Bay, ME (ME), Harbor Cove, MA (HC), Cape Cod, MA (CC), Orient Harbor, NY (OH), Raritan Bay, NY (RB), Great Bay, NJ (GB), Atlantic City, NJ (AC), Assateague, MD (AT), Wachapreague, VA (WP), James River, VA (JR), Mobjack Bay, VA (MB), Pocomoke Sound, VA (PS), Bogue Sound, NC (BS), and North Inlet, SC (NI). Inset: MD and VA sampling locations. Major geographic landmarks indicated with text and identified genetic breaks marked with dashed lines.

Hard clam samples (*n* = 452 total) were collected from wild populations from 15 sites along the east coast of North America from Prince Edward Island, Canada, to North Inlet, SC, USA, with more intensive sampling in the Mid‐Atlantic region, including Chesapeake Bay, Virginia. Sampling locations included: Prince Edward Island, Canada (PI), Middle Bay, ME (ME), Harbor Cove, MA (HC), Cape Cod, MA (CC), Orient Harbor, NY (OH), Raritan Bay, NY (RB), Great Bay, NJ (GB), Atlantic City, NJ (AC), Assateague, MD (AT), Wachapreague, VA (WP), James River, VA (JR), Mobjack Bay, VA (MB), Pocomoke Sound, VA (PS), Bogue Sound, NC (BS), and North Inlet, SC (NI) (Table 1, Figure 1).

**TABLE 1:** List of hard clam collection sites with number of individual clams that were collected from each location and genetic diversity statistics by sampling location; expected heterozygosity (*H*_e_), observed heterozygosity (*H*_o_), and inbreeding coefficient (*G*_IS_).

Location	State	Loc. ID	Avg. height	Year (if lethal sample)	Number sent for DArTseq™	*H*_e_	*H*_o_	*G*_IS_
Prince Edward Island	Canada	PI	44.5	2019	32	0.186	0.172	0.092
Maine	ME	ME	44.6	2019	32	0.199	0.185	0.087
Harbor Cove	MA	HC	N/A	2012	32	0.198	0.171	0.151
Cape Cod	MA	CC	N/A	Non‐lethal	30	0.199	0.172	0.151
Orient Harbor	NY	OH	81.4	2019	32	0.197	0.171	0.145
Raritan Bay	NY	RB	59.7	2019	31	0.195	0.174	0.123
Great Bay	NJ	GB	N/A	Non‐lethal	24	0.194	0.172	0.132
Atlantic City	NJ	AC	56.6	2019	31	0.195	0.176	0.113
Assateague	MD	AT	69.5	2019	32	0.195	0.173	0.129
Wachapreague	VA	WP	81.1	2018	31	0.193	0.169	0.139
Pocomoke Sound	VA	PS	N/A	Non‐lethal	20	0.191	0.171	0.121
Mobjack Bay	VA	MB	69.0	2018	31	0.193	0.171	0.130
James River	VA	JR	53.6	2018	32	0.193	0.173	0.132
Bogue Sound	NC	BS	70.4	2018	31	0.190	0.167	0.135
North Inlet	SC	NI	59.5	2018	31	0.191	0.167	0.139
Total					452			

*Note*: *H*_o_ was significantly lower than *H*_e_ in all samples (*p* < 0.0001).

Tissue samples were either taken lethally from the foot and adductor muscle of each clam or mantle tissue was nonlethally sampled from clams being held as brood stock at the Virginia Institute of Marine Science Eastern Shore Laboratory. Foot and adductor tissues were stored in 95% ethanol at −20°C and mantle tissues were stored in RNAlater™ (Invitrogen) at −20°C. Genomic DNA was isolated from foot and mantle tissue using the DNeasy Blood & Tissue Kit (Qiagen) with the optional RNase A treatment as per the manufacturer's protocol. DNA quantity and purity were assessed using the NanoDrop Spectrophotometer (NanoDrop Technologies Inc.) and quality was assessed by horizontal agarose gel electrophoresis of samples alongside a 1 Kb Plus DNA ladder (Invitrogen).

### 2.2 Next‐generation sequencing

To identify SNP loci, isolated DNA was sent to Diversity Arrays Technology (DArT Pty Ltd) at concentrations between 50 and 100 ng/μL for high‐throughput GBS using DArTseq™ (Georges et al., 2018; Kilian et al., 2012; Sansaloni et al., 2011). Approximately 30 individuals from each of the 15 sampling locations were chosen for DArTseq™ to avoid ascertainment bias and to accurately capture the genetic variation at each sampling location. The DArTseq™ method includes a restriction enzyme digestion step to reduce genome complexity followed by hybridization to microarrays and massively parallel sequencing followed by SNP identification (Sansaloni et al., 2011).

### 2.3 Data filtering

Sequenced SNP data were filtered to minimize genotyping errors and missing data using the R software (R Core Team, 2018) package *dartR* v1.3.4 (Gruber et al., 2018) (Table S1). Loci with less than 98% reproducibility were removed (Georges et al., 2018). To address the proportion of missing data, call rate was set at <98% for loci and at <95% for individuals. Data were first filtered for call rate by locus to retain as many individuals as possible in the analysis. Data were also filtered to remove loci based on Hamming distance (<20%) (Hamming, 1950; Pilcher et al., 2008). When more than one SNP was found within the same 69 base pair sequence fragment (i.e., a secondary SNP), one was retained at random to ensure that loci were independent. Loci with a minor allele frequency (MAF) of less than 1% were removed and data were also filtered for read depth with thresholds set to remove loci with less than 5× coverage depth or more than 75× coverage to minimize the likelihood of errors in the data.

After SNP data were filtered in *dartR*, additional filtering was applied to identify loci that deviated from the expectations of Hardy–Weinberg equilibrium (HWE) in multiple sampling locations, likely because of genotyping errors and other technical artifacts, and to compare individual levels of average heterozygosity across loci to look for evidence of cross‐contamination in the R package *radiator* v1.1.4 (Gosselin et al., 2020). Loci that did not conform to the expectations of HWE in at least four sampling locations using a *p*‐value threshold of 0.01 were removed from the dataset.

### 2.4 Identification of outlier loci

Outlier loci were identified by two different methods: *OutFLANK* (Lotterhos & Whitlock, 2015), which was run in the R package *dartR*, and using the R package *pcadapt* v4.3.3 (Privé et al., 2020). The default parameters were used for *OutFLANK*. For *pcadapt*, the default threshold of minor allele frequency (MAF) was used, and the false discovery rate (FDR) method was implemented, with loci having *q*‐values less than the FDR of α set to 10% considered outliers. The outliers identified by both *OutFLANK* and *pcadapt* were compared and the subset of loci identified by both methods was retained. Therefore, along with the “full dataset,” two additional datasets were created; the “outlier dataset” included only the loci identified using both methods, and the “neutral dataset,” which excluded those loci included in the outlier dataset. All three datasets were analyzed as described below. BLAST searches (Altschul et al., 1990) were conducted for the outlier dataset using MegaBlast on the NCBI website to optimize for highly similar sequences.

### 2.5 Data analysis: Genetic diversity and population structure based on all datasets

The filtered datasets were used to estimate levels of genetic diversity within collections from different geographic locations and the level of divergence among collections. Observed heterozygosity (*H*_o_) and expected heterozygosity (gene diversity, *H*_e_) were calculated in the R package *adegenet* v2.1.2 (Jombart, 2008; Jombart & Ahmed, 2011), and the coefficient of inbreeding (*G*_IS_, Nei, 1987) was calculated in genodive v3.03 (Meirmans, 2020). The level of genetic differentiation among sample collections was assessed using unbiased *F*‐statistics (*F*_ST_, Weir & Cockerham, 1984). Pairwise significance was based on 10,000 iterations of the data.

Several alternative methods with differing underlying assumptions were used to delineate population structure. A principal component analysis (PCA) was used to visually explore the population structure and the level of genetic differentiation among clams within and between each sampling location using *adegenet*, which calculates Euclidean genetic distances between samples (Jombart et al., 2009). Samples were grouped into genetically distinct populations using a Bayesian model‐based clustering method implemented in structure v2.3.4 (Pritchard et al., 2000). Cluster analysis was conducted on the complete filtered dataset and, due to the uniqueness of the Prince Edward Island samples, on a subset of the data excluding the Prince Edward Island samples (hereafter the “no PI” subset) to resolve finer‐scale (more nested) population structure obscured by their inclusion (Janes et al., 2017). Both datasets were run with the admixture model and sampling locations were used as prior information, the latter of which can help with identifying structure when levels of divergence are lower (Hubisz et al., 2009). Allele frequencies were allowed to be correlated between locations to assist in identification of closely related populations (Falush et al., 2003). Each dataset was run with 10 replicates per *K* value, *K* being the inferred number of ancestral lineages: *K* = 1–7 for the full dataset and *K* = 3–6 for the no PI subset. Initially, the range of *K* values tested for each dataset corresponded to the number of sampling locations included. The most supported *K* values from those runs informed the final range of *K* values tested. Each replicate had a burn‐in of 500,000 Markov Chain Monte Carlo (MCMC) iterations followed by 500,000 MCMC iterations. Results were compiled in Structure Harvester (Earl & vonHoldt, 2012) to identify the most well‐supported *K* value based on the Evanno method (Evanno et al., 2005). Replicates were combined and visualized using the R package *pophelper* v2.3.0 (Francis, 2017). A discriminant analysis of principal components (DAPC) (Jombart et al., 2010), which does not assume HWE, was also used to assess the most likely number of clusters comprised by the data in *adegenet* for both of the datasets described above. The optimal number of PCs retained for de novo cluster identification and DAPC analysis was determined using the function *xvalDapc*. The function *find*. *clusters* was first run to assess increasing numbers of clusters (*K* = 1 to *K* = 15) to identify de novo the optimal number of clusters based on the Bayesian information criterion (BIC) value, and individuals were assigned to these clusters with the DAPC.

To further address partitioning of sampling locations into different genetic populations, analysis of molecular variance (AMOVA, Excoffier et al., 1992; Michalakis & Excoffier, 1996) was conducted in genodive (Meirmans, 2020) with the full dataset. Several alternative hypotheses of hierarchical population structure were tested using the infinite alleles model with statistics corresponding to Weir and Cockerham's (1984) *F*‐statistics. Four alternate scenarios were tested with sampling locations grouped in increasing numbers of genetic clusters (Table 2). Significance was based on 10,000 iterations of the data.

**TABLE 2:** Results of the AMOVA analyses for each of the four AMOVA groupings including the percent of variation explained, the *F*‐statistic, and corresponding *p*‐values.

Source of variation	% Variation	*F*‐statistic	*p*‐value
AMOVA grouping 1 [PI] [all other locations]			
Within individuals	0.838	*F*_IT_ = 0.162	–
Among individuals, within sites	0.122	*F*_IS_ = **0.127**	0.0001
Among sites, within groups	0.006	*F*_SC_ = **0.006**	0.0001
Among groups	0.034	*F*_CT_ = **0.034**	0.0001
AMOVA grouping 2 [PI] [ME] [CC, HC] [OH‐NI]			
Within individuals	0.859	*F*_IT_ = 0.141	–
Among individuals, within sites	0.125	*F*_IS_ = **0.127**	0.0001
Among sites, within groups	0.001	*F*_SC_ = **0.001**	0.0001
Among groups	0.014	*F*_CT_ = **0.014**	0.0001
AMOVA grouping 3 [PI] [ME] [CC, HC] [OH‐WP] [PS, MB, JR] [BS, NI]			
Within individuals	0.861	*F*_IT_ = 0.139	–
Among individuals, within sites	0.126	*F*_IS_ = **0.127**	0.0001
Among sites, within groups	0.000	*F*_SC_ = 0.000	0.0562
Among groups	0.013	*F*_CT_ = **0.013**	0.0001
AMOVA grouping 4 [PI] [ME] [CC, HC] [OH] [RB‐WP] [PS, MB, JR] [BS, NI]			
Within individuals	0.862	*F*_IT_ = 0.138	–
Among individuals, within sites	0.126	*F*_IS_ = **0.127**	0.0001
Among sites, within groups	0.000	*F*_SC_ = 0.000	0.6968
Among groups	0.012	*F*_CT_ = **0.012**	0.0001

*Note*: Each AMOVA was run for 10,000 iterations with the infinite alleles model. The *F*‐statistic is bolded if significant genetic differentiation is explained among sources of variation compared.

### 2.6 Data analysis: Diversity and divergence of genetic populations

Once population structure had been delineated, the levels of genetic diversity within populations and the level of divergence among populations including *H*_o_, *H*_e_, *G*_IS_, and *F*_ST_ were calculated as above. Pairwise significance of *F*_ST_ values was based on 10,000 iterations of the data. The effective population size (*N*_e_) of each genetic population was determined to assess the impacts of genetic drift and inbreeding on the populations using NeEstimator v2.1 (Do et al., 2014). The linkage disequilibrium (LD) model was used with random mating and assessed with 95% confidence intervals of three critical values (allele frequencies); 0.01, 0.02, and 0.05.

To determine if the data were consistent with a pattern of isolation by distance (IBD), a Mantel test (Mantel, 1967) of the correlation between genetic and geographic distance matrices was conducted in *adegenet*. IBD can be confounded by population structure and spatial autocorrelation (Meirmans, 2012), so IBD was assessed in the Mid‐Atlantic region between the genetic breaks previously identified at Cape Cod, MA, and Cape Hatteras, NC (i.e., for samples collected between New York and Virginia) (Baker et al., 2008). Nei's genetic distance (Nei, 1972, 1978) between populations was calculated in *adegenet*. Geographic distance was measured in Google Maps with the shortest overwater distance between locations from best‐known coordinates of sampling locations. The significance of the correlation was assessed with 50,000 permutations of the data.

## 3 RESULTS

### 3.1 Filtering and construction of datasets

DArTseq™ of the complete sample set (*n* = 452, Table 1) identified 153,842 SNP loci. The final filtered dataset contained 448 individuals from 15 locations and 4960 SNPs (Table S1). Average heterozygosity of individuals ranged from 0.147 to 0.252, with a sample mean heterozygosity of 0.172. There was no evidence of cross‐contamination of samples based on heterozygosity values and no individuals were removed from the dataset.

There were 184 loci identified as outliers in *OutFLANK* and 142 loci identified as outliers in *pcadapt*. Overall, 95 loci were identified by both methods and comprised the final outlier dataset. This resulted in three datasets: all loci (4960), neutral loci (4865), and outlier loci (95), with the samples from Prince Edward Island removed (no PI dataset) for some analyses. PI samples were removed from the datasets when they obscured lower levels of population structure. Comparison of results of the analyses that included all loci and the analyses that included only neutral loci were congruent, therefore only the results based on all loci and outlier loci datasets, with and without PI, are presented below.

### 3.2 Diversity and divergence among sampling locations—All loci

When calculated for each sampling location separately, gene diversity (*H*_e_) generally increased with latitude except for the PI sample, which had the lowest *H*_e_ (0.186). For all sampling locations, observed heterozygosity was significantly lower than expected heterozygosity (*H*_o_: 0.167–0.185, *H*_e_: 0.186–0.199, *p* < 0.001) (Table 1).

Pairwise *F*_ST_ values ranged from 0 to 0.047 (Table 3). The highest level of divergence was found in comparisons between Prince Edward Island and every other sampling location (*F*_ST_ = 0.032–0.047) where all comparisons were significantly different (*p* < 0.0001). Middle Bay, ME, was also significantly different from all other sampling locations (*F*_ST_ = 0.006 vs. HC–0.032 vs. PI, *p* < 0.0001). Samples collected from the two locations in Massachusetts (HC and CC) were not significantly different from each other (*F*_ST_ = 0.000, *p* = 0.493) but were significantly different from samples from all other sampling locations (HC: *F*_ST_ = 0.003 vs. GB–0.035 vs. PI, CC: *F*_ST_ = 0.003 vs. GB–0.038 vs. PI, *p* < 0.0001). Samples from locations between Cape Cod and Cape Hatteras had varying levels of differentiation. Clams from Orient Harbor, NY, were not significantly different from Great Bay, NJ (*F*_ST_ = 0.001, *p* = 0.101) but were significantly different from all other locations (*F*_ST_ = 0.001–0.040, *p* = 0.001–0.0084). Clams ranging between Raritan Bay, NY, and Wachapreague, VA (RB, GB, AC, AT, and WP) were not significantly different from each other (*F*_ST_ = 0–0.001, *p* = 0.064–0.854). Samples from the three locations within the Chesapeake Bay, VA, were not significantly different from each other (*F*_ST_ = −0.001–0.000, *p* = 0.356–0.852) but all were significantly different from all other samples including clams collected from Wachapreague on the oceanside of Virginia (*F*_ST_ = 0.001–0.044, *p* = 0.001–0.018). Samples from the two southernmost locations collected south of Cape Hatteras in the Carolinas (BS and NI) were not significantly different from each other (*F*_ST_ = 0, *p* = 0.890) but were significantly different from samples from all other sampling locations (BS: *F*_ST_ = 0.007 vs. WP and JR–0.047 vs. PI, NI: *F*_ST_ = 0.005 vs. WP–0.045 vs. PI, *p* < 0.0001).

**TABLE 3:** Pairwise *F*_ST_ values of the total dataset between sampling locations (lower matrix) and *p*‐values (upper matrix) are based on 10,000 iterations of the data.

	PI	ME	HC	CC	OH	RB	GB	AC	AT	WP	JR	MB	PS	BS	NI
PI		**0.000**	**0.000**	**0.000**	**0.000**	**0.000**	**0.000**	**0.000**	**0.000**	**0.000**	**0.000**	**0.000**	**0.000**	**0.000**	**0.000**
ME	**0.032**		**0.000**	**0.000**	**0.000**	**0.000**	**0.000**	**0.000**	**0.000**	**0.000**	**0.000**	**0.000**	**0.000**	**0.000**	**0.000**
HC	**0.035**	**0.006**		0.493	**0.000**	**0.000**	**0.000**	**0.000**	**0.000**	**0.000**	**0.000**	**0.000**	**0.000**	**0.000**	**0.000**
CC	**0.038**	**0.008**	0.000		**0.000**	**0.000**	**0.000**	**0.000**	**0.000**	**0.000**	**0.000**	**0.000**	**0.000**	**0.000**	**0.000**
OH	**0.040**	**0.012**	**0.004**	**0.004**		**0.008**	0.101	**0.002**	**0.000**	**0.000**	**0.000**	**0.000**	**0.000**	**0.000**	**0.000**
RB	**0.042**	**0.012**	**0.004**	**0.004**	**0.001**		0.472	0.848	0.579	0.129	**0.000**	**0.000**	**0.000**	**0.000**	**0.000**
GB	**0.041**	**0.013**	**0.003**	**0.003**	0.001	0.000		0.854	0.682	0.678	**0.001**	**0.000**	**0.000**	**0.000**	**0.000**
AC	**0.040**	**0.012**	**0.004**	**0.004**	**0.002**	−0.001	−0.001		0.354	0.094	**0.000**	**0.000**	**0.000**	**0.000**	**0.000**
AT	**0.043**	**0.013**	**0.004**	**0.005**	**0.002**	0.000	0.000	0.000		0.064	**0.000**	**0.000**	**0.000**	**0.000**	**0.000**
WP	**0.041**	**0.013**	**0.004**	**0.005**	**0.003**	0.001	0.000	0.001	0.001		**0.001**	**0.018**	**0.004**	**0.000**	**0.000**
JR	**0.043**	**0.015**	**0.006**	**0.008**	**0.006**	**0.003**	**0.002**	**0.003**	**0.002**	**0.002**		0.618	0.356	**0.000**	**0.000**
MB	**0.044**	**0.016**	**0.006**	**0.007**	**0.005**	**0.003**	**0.003**	**0.002**	**0.002**	**0.001**	0.000		0.852	**0.000**	**0.000**
PS	**0.043**	**0.015**	**0.007**	**0.007**	**0.005**	**0.004**	**0.003**	**0.004**	**0.003**	**0.002**	0.000	−0.001		**0.000**	**0.000**
BS	**0.047**	**0.020**	**0.010**	**0.012**	**0.011**	**0.008**	**0.009**	**0.009**	**0.008**	**0.007**	**0.007**	**0.008**	**0.008**		0.890
NI	**0.045**	**0.019**	**0.010**	**0.011**	**0.011**	**0.008**	**0.008**	**0.008**	**0.007**	**0.005**	**0.006**	**0.007**	**0.007**	−0.001	

*Note*: *F*_ST_ values that are significant are bolded. Pairwise *F*_ST_ values are shaded based on degree of differetiation, with highest differentiation shown in the darkest grey.

**FIGURE 2:**
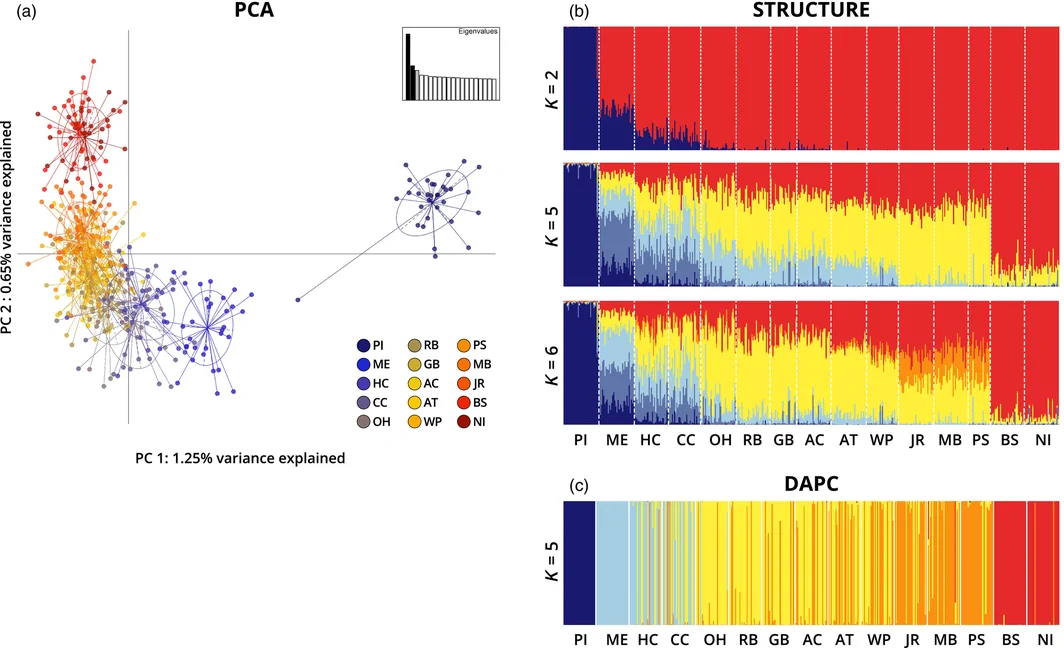
(a) PCA of PC 1 versus PC 2 of the total dataset totaling 1.9% of variance explained. (b) structure plot of all locations, *K* = 2, 5, and 6. Each *K* was run with 500,000 burn‐in, followed by 500,000 iterations and 10 replicates each. The admixture model was used, with sampling locations as a prior, and allele frequencies correlated. (c) DAPC with the most optimal clustering based on BIC, *K* = 5. Prince Edward Island, Canada (PI), Middle Bay, ME (ME), Harbor Cove, MA (HC), Cape Cod, MA (CC), Orient Harbor, NY (OH), Raritan Bay, NY (RB), Great Bay, NJ (GB), Atlantic City, NJ (AC), Assateague, MD (AT), Wachapreague, VA (WP), James River, VA (JR), Mobjack Bay, VA (MB), Pocomoke Sound, VA (PS), Bogue Sound, NC (BS), and North Inlet, SC (NI).

The PCA of all loci resulted in 1.25% variance explained by principal covariate 1 (PC 1), 0.65% by PC 2, and 0.56% by PC 3 (Figure 2a). The largest separation on PC 1 was between PI clams and those from all other sampling locations. Based on PC 1 versus PC 2, clams from all sampling locations except PI showed a latitudinal gradient of separation. Many of the sampling locations did not separate completely from each other but were generally clustered by geographic region.

Cluster‐based assessment of population structure using structure and DAPC produced similar results (Figure 2b,c). When all geographic sampling locations were included in the analysis, *K* = 2 clusters were the most supported, structure separating clams sampled from PI from clams sampled from all other sampling locations, although ME and MA samples showed mixed ancestry (Figure 2b). Additionally, *K* = 5 and *K* = 6 were also well supported and distinguished the following groups: (1) PI, (2) ME, (3) MA (HC and CC), (4) Mid‐Atlantic (OH to WP), (5) the Chesapeake Bay (especially with *K* = 6; JR, MB, and PS), and (6) the Carolinas (BS & NI). When the PI sample was excluded from analyses, the most supported numbers of clusters, *K* = 3 and *K* = 5, showed a similar pattern (Figure S1). DAPC analysis of all loci indicated that the optimal number of PCs to retain was 50, and the BIC indicated that the optimal number of clusters was *K* = 5 (Figure 2c, Figure S2). In the DAPC that included all sampling locations, the groupings identified were the same as those identified using STRUCTURE (Figure 2b,c). Exclusion of PI from analysis did not result in increased resolution (Figure S3). Hierarchical AMOVAs indicated a significant difference among sampling locations within groups (*F*_SC_) until six groups were tested (Table 2). The optimal grouping was consistent with the groupings delineated by the individual‐based clustering methods (*F*_CT_ = 0.013, *p* < 0.0001) (Table 2).

### 3.3 Diversity and divergence of genetic populations—All loci

Diversity generally increased with latitude except for the finding of lower diversity in the Canadian population. Excluding the Canadian population, which had a *H*_e_ of 0.186 and *H*_o_ of 0.172, *H*_e_ ranged from 0.200 in Massachusetts to 0.192 in North Carolina and *H*_o_ ranged from 0.185 in Maine to 0.167 in North Carolina with observed heterozygosity values significantly lower than expected heterozygosity values (*p* < 0.0001) (Table 4). For all populations, *G*_IS_ ranged from 0.087 (Maine) to 0.151 (Massachusetts) (Table 4). All genetic populations were significantly different from each other based on pairwise *F*_ST_ values (Table 5), with the largest difference being between Canada and the Carolinas (*F*_ST_ = 0.046, *p* < 0.0001), and the smallest difference was between the Chesapeake Bay and the Mid‐Atlantic (*F*_ST_ = 0.003, *p* < 0.0001).

**TABLE 4:** Genetic diversity statistics by genetic populations; expected heterozygosity (*H*_e_), observed heterozygosity (*H*_o_), and inbreeding coefficient (G_IS_) and estimated effective population size (*N*_e_) with 95% confidence intervals using three different allele frequencies 0.01, 0.02, and 0.05 with the linkage disequilibrium method.

Genetic population	*H*_e_	*H*_o_	*G*_IS_	*N*_e_ (95% CI)		
				Allele Freq. 0.01	Allele Freq. 0.02	Allele Freq. 0.05
Canada	0.186	0.172	0.092	1176.5 (1043.2–1348.4)	1148.6 (1007.5–1335.1)	1033.2 (896.6–1218.2)
Maine	0.199	0.185	0.087	626.4 (590.4–667.0)	460.7 (438.2–485.5)	517.5 (482.9–557.3)
Massachusetts	0.200	0.172	0.151	7837.7 (5690.9–12,572.2)	6790.5 (5000.4–10,568.3)	5582.2 (4082.1–8813.5)
Mid‐Atlantic	0.198	0.173	0.13	10,455.4 (9072.5–12,332.8)	8702.1 (7595.5–10,184.0)	6860.3 (5953.1–8090.9)
Chesapeake Bay	0.195	0.172	0.128	17,592.4 (11,127.8–41,923.0)	9809 (7141.5–15,641.7)	6335.8 (4808.3–9277.0)
Carolinas	0.192	0.167	0.137	19,796.2 (12,176.3–352,970.2)	16,103.5 (8736.1–101,909.6)	27,814.9 (9512.9–Infinite)

*Note*: *H*_o_ was significantly lower than *H*_e_ in all populations (*p* < 0.0001).

**TABLE 5:** Pairwise *F*_ST_ values of the total dataset between genetic populations (lower matrix), and *p*‐values (upper matrix) are based on 10,000 iterations of the data.

	Canada	Maine	Massachusetts	Mid‐Atlantic	Chesapeake Bay	Carolinas
Canada		0.0001	0.0001	0.0001	0.0001	0.0001
Maine	0.032		0.0001	0.0001	0.0001	0.0001
Massachusetts	0.035	0.007		0.0001	0.0001	0.0001
Mid‐Atlantic	0.040	0.012	0.004		0.0001	0.0001
Chesapeake Bay	0.042	0.015	0.007	0.003		0.0001
Carolinas	0.046	0.020	0.011	0.008	0.007	

Pairwise *F*_ST_ values are shaded based on degree of differetiation, with highest differentiation shown in the darkest grey.

Estimated effective population size (*N*_e_) for all populations at all assessed allele frequency categories ranged from 461 in Maine to 27,815 in the Carolinas (Table 4). There was a general decrease in estimated *N*_e_ with increasing latitude. Maine clams consistently had the lowest estimated *N*_e_ across all allele frequencies ranging from 461 to 626 and the Carolina clams consistently had the highest estimated *N*_e_ ranging from 16,104 to 27,815. Canadian clams had an estimated *N*_e_ that was higher than Maine clams, but lower than all other groups, ranging from 1033 to 1177. Estimated *N*_e_ for Massachusetts, Mid‐Atlantic, and Chesapeake Bay clams were more like each other than the other sampled populations, with Massachusetts' range being slightly lower (Massachusetts: 5583–7838, Mid‐Atlantic: 6860–10,455, and Chesapeake Bay: 6336–17,592).

Mantel tests of isolation by distance for locations between the genetic breaks identified at Cape Cod and Cape Hatteras (OH to PS) indicated a significant signal of IBD in the Mid‐Atlantic region. There was a positive correlation between Nei's genetic and geographic distance matrices; *r* = 0.49, *p* = 0.039 (Figure S4).

### 3.4 Genetic structure and divergence—Outlier loci

**FIGURE 3:**
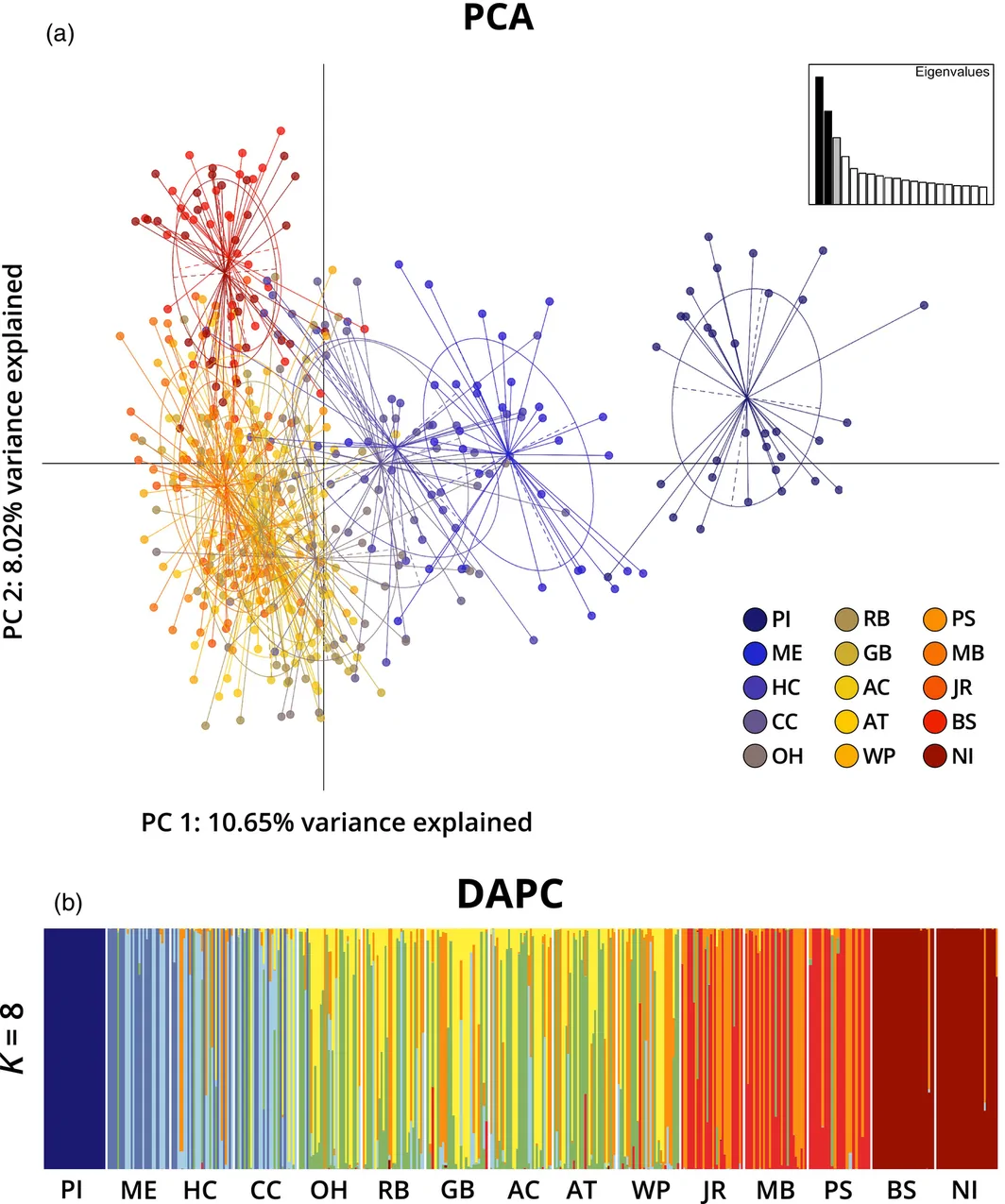
(a) PCA of PC 1 versus PC 2 of the outlier dataset totaling 18.67% of variance explained. (b) DAPC of outlier loci with the most optimal clustering based on BIC, *K* = 8. Prince Edward Island, Canada (PI), Middle Bay, ME (ME), Harbor Cove, MA (HC), Cape Cod, MA (CC), Orient Harbor, NY (OH), Raritan Bay, NY (RB), Great Bay, NJ (GB), Atlantic City, NJ (AC), Assateague, MD (AT), Wachapreague, VA (WP), James River, VA (JR), Mobjack Bay, VA (MB), Pocomoke Sound, VA (PS), Bogue Sound, NC (BS), and North Inlet, SC (NI).

BLAST searches on the 95 outlier loci found 29 significant matches, 15 of which were uncharacterized transcripts, however, proteins related to those involved in stress response, immune function, protein processing, and regulation, such as heat shock, zinc finger‐like, complement C3, and calmodulin proteins, were identified (Table S2). A PCA of the dataset comprised of the outlier loci showed 10.96% variance explained by PC 1, 8.02% by PC 2, and 5.75% by PC 3 (Figure 3a). The clustering pattern was comparable to that observed in the PCA that included all loci, with more distinct separation and a higher percentage of the variance explained. DAPC analysis of outlier loci indicated that 70 PCs were the most optimal to retain for analysis and *K* = 8 was the most optimal number of clusters based on comparison of BIC values (Figure S5). When sampling locations were grouped based on similarity of the proportions of different clusters, they were comparable to the results for the total dataset except that a more distinct break was observed between samples from the ocean side of Virginia (WP) and those located within the Chesapeake Bay (JR, MB, and PS) (Figure 3b).

Pairwise *F*_ST_ values among locations for the outlier dataset ranged from 0 to 0.325 (Table S3). Locations that were significantly different in *F*_ST_ analysis of both the total dataset and the outlier dataset had *F*_ST_ values that were an order of magnitude higher based on the outlier dataset (Total *F*_ST_ = 0.001–0.047; Outlier *F*_ST_ = 0.01–0.325). Pairwise comparisons between some locations that were not significantly different based on analysis with the full dataset were significantly different from each other using the outlier dataset including HC and CC (*F*_ST_ = 0.011, *p* = 0.0099), OH and GB (*F*_ST_ = 0.014, *p* = 0.0073), and WP and RB, GB, and AC (*F*_ST_ = 0.01–0.013, *p* = 0.0088–0.0254).

## 4 DISCUSSION

### 4.1 Population structure

This study employed a high‐resolution set of SNP markers to describe the population structure of the hard clam, *Mercenaria mercenaria*, across a large portion of its native range. Samples from 15 locations from PI to SC resulted in delineation of 6 genetically distinct populations based on analyses with 4960 SNP loci. The five genetic breaks identified by the whole dataset were also recovered by the outlier dataset, increasing the resolution of previous studies based on fewer genetic markers and sampling locations (Baker et al., 2008; Dillon & Manzi, 1992).

It is common for species that occupy bays or estuaries to have abrupt genetic discontinuities (Miller et al., 2006), likely due to shifting ocean currents and landmasses acting as barriers to gene flow. The geographic distribution of the hard clam populations identified by this study largely corresponds to the marine biogeographic provinces that shape the western North Atlantic (Briggs & Bowen, 2012). The northernmost Canadian population consisting of clams sampled from Prince Edward Island (PI) most diverged from all other populations examined. The elevated level of divergence suggests that the eastern Nova Scotian shelf is a significant geographic barrier for the hard clam. The eastern Nova Scotian shelf region separates the Gulf of St. Lawrence from the Labrador Current and has been identified as a genetic boundary for a wide range of marine species including the American lobster (*Homarus americanus*), Atlantic cod (*Gadus morhua*), sea scallop (*Placopecten magellanicus*), northern shrimp (*Pandalus borealis*), European green crab (*Carcinus maenas*), and the Atlantic silverside (*Menidia menidia*) (Benestan et al., 2015; Mach et al., 2011; Stanley et al., 2018).

The hard clam populations that were resolved along the US east coast in this study were separated by genetic barriers identified between (1) Maine and Massachusetts, (2) Cape Cod, (3) between the bayside and oceanside of the Delmarva Peninsula, and (4) Cape Hatteras. The observed genetic discontinuities were most pronounced when comparing samples across Cape Hatteras and Cape Cod—two barriers known to disrupt gene flow in other marine species (Mach et al., 2011). These capes define the three biogeographic provinces that are characterized by their temperature, salinity, and seasonality: the Acadian, the Virginian, and the Carolinian (Hale, 2010; Mach et al., 2011). The Acadian province north of Cape Cod has water that is cold and stable year‐round, influenced by the Labrador Current and the Gulf of Maine. The Virginian province has higher, seasonally variable water temperatures, and to the south of Cape Hatteras, the Carolinian province is characterized by the warmer‐temperate waters that are carried north by the Gulf Stream.

A genetic break was also identified between Maine and Massachusetts, which is bounded by the Nova Scotian shelf to the north and Cape Cod to the south. Bayesian model‐based clustering indicated that Maine clams had a different relative proportion of northern and southern ancestry than Massachusetts clams (Figure 2b,c). Although the observed genetic discontinuity between Maine and Massachusetts does not correspond to a well‐known biogeographic break, Dillon and Manzi (1992) also found significant genetic differences between Maine and Massachusetts hard clam samples. Despite the observed difference, they concluded that hard clams from Maine and Massachusetts comprised a single fisheries stock because the magnitude of differentiation observed was low and there was no evident barrier to gene flow. However, the distribution of wild hard clams from Cape Cod to the Gulf of St. Lawrence has been noted to be patchy, with habitat discontinuity in the Gulf of Maine (Baker et al., 2008; MacKenzie et al., 2001). This habitat discontinuity could affect larval survival and population connectivity in the region, resulting in smaller, more isolated, populations. This is consistent with the small *N*_e_ estimate of the Maine population. The use of a comparatively high number of markers in the current study resulted in resolution of two genetically distinct populations in this region: Maine and Massachusetts.

In some cases, outlier loci are suspected to be adaptive under divergent natural selection and resolve finer‐scale and unique population structure as compared to neutral markers (Batista et al., 2016; Hargrove et al., 2015). However, observed differences could also be an artifact of demographic processes including allele surfing during range expansions, which can result in a rapid drift of some alleles at the leading edge of the expansion (reviewed in Hoban et al., 2016; Travis et al., 2007). In the current study, there was an order‐of‐magnitude difference in divergence values between neutral and outlier loci, but the patterns of structure between the two datasets were concordant in that both recovered six major genetic populations. The congruence between the two datasets and the life history characteristics of the hard clam suggests that neutral processes are more likely responsible for the observed differences between the two datasets.

### 4.2 Fine‐scale genetic structure

To address genetic structure in the Mid‐Atlantic region, which lacked representation in previous studies (e.g., Baker et al., 2008), more intensive sampling was carried out between Cape Cod and Cape Hatteras. Results from both the full dataset and outlier dataset indicate the presence of two distinct genetic populations: the Mid‐Atlantic, comprised of coastal ocean samples from New York‐Virginia (OH‐WP), and the Chesapeake Bay, comprised of samples taken within Chesapeake Bay (JR, MB, PS). The finding of fine‐scale population structuring between hard clams sampled from Chesapeake Bay and Mid‐Atlantic locations on the oceanside is consistent with a phylogeographic boundary and intraspecies transition as the high salinity waters of the Atlantic Ocean (average ~32 ppt) and the lower salinity estuarine waters of Chesapeake Bay (average range from sampling sites ~15–25 ppt) (www.chesapeakebay.net) are separated by the Delmarva Peninsula. Optimum salinity for the hard clam is between 20 and 38 parts per thousand (ppt), but they can survive in salinities as low as 12.5 (Castagna & Chanley, 1973; Castagna & Kraeuter, 1981; Mulholland, 1984). Hard clams originating from the bayside and the oceanside of the Virginian Eastern Shore also show regional differences in growth, with those in the estuary growing more slowly. A meta‐analysis based on the mitochondrial COI gene that included the data for *M. mercenaria* from Dillon and Manzi (1987) and Baker et al. (2008) along with data from many other species found evidence of the Delmarva‐associated genetic break not only in *M. mercenaria* but also in several other species with long pelagic larval phases including *Marenzelleria viridis*, *Geukensia demissa*, *Melampus bidentatus*, *Uca pugilator*, and *Fundulus heteroclitus* (Altman et al., 2013).

In marine systems, outlier loci have been shown to better differentiate fine‐scale genomic patterns and provide greater resolution for population divergence than neutral loci when population structure is weak (Hohenlohe et al., 2018). Analysis with the outlier dataset showed a comparatively small but significant difference (*F*_ST_ values) between samples from the two Massachusetts locations, as well as between samples from locations in the Mid‐Atlantic that were not observed with the dataset that included all loci (Table S3) including between Wachapreague (WP) and several other locations (RB, GB, and AC), and between OH and GB. This subtle difference between the two datasets could be attributed to the outlier loci being adaptive, as discussed above. BLAST searches indicated that some outlier loci identified in this study had matches to proteins involved in stress response, immune function, protein processing, and regulation, indicating that they might be involved in responses to environmental stressors. However, the additional structure suggested by the outlier dataset could also be caused by random stochastic processes including human movement of clam stocks and sweepstakes reproduction. Additional studies, including comparative transcriptomic studies and population genetic studies that include environmental data, will be needed to definitively resolve whether selection is playing a role in the maintenance of population structure in the hard clam.

### 4.3 Isolation by distance

For sampling locations between Cape Cod and Cape Hatteras, including the Chesapeake Bay and Mid‐Atlantic populations, there was a significant correlation between genetic distance and geographic distance. This pattern of IBD could indicate that the sampling locations are connected through larval dispersal, likely from north to south via the Virginia coastal current (Wares, 2002). While the hard clam has a long pelagic larval phase that could theoretically facilitate connectivity between these locations, the finding of a pattern of IBD in this region, as opposed to a panmictic population, indicates a shorter realized dispersal and connectivity among hard clams in close geographic proximity. Patterns of IBD have been identified in other bivalves in the region, including *C. virginica* within the Chesapeake Bay (Rose et al., 2006). The relative genetic isolation of species within western North Atlantic coastal bays has also been noted for bay scallops (Bert et al., 2011; Wilbur et al., 2005), and as a constraint to recolonization of seagrasses following catastrophic loss (Orth et al., 2020).

### 4.4 Levels of genetic divergence and genetic diversity

The pairwise *F*_ST_ levels among hard clam populations in this study were low overall but revealed statistically significant levels of divergence, a pattern that has been observed in other invertebrates with a pelagic larval phase whose population structure was examined using genotyping by sequencing. For example, Benestan et al. (2015) found hierarchical population structure for the American lobster, *Homarus americanus*, by first identifying a northern and southern divide along the Scotian shelf (*F*_CT_ = 0.0011; *p* = 0.0002) and then identifying fine‐scale structuring of 11 putative genetic populations of lobster from 17 sampling locations (mean *F*_ST_ = 0.00185; CI: 0.0007–0.0021, *p* < 0.0002). A study of the giant California sea cucumber (*Parastichopus californicus*) also revealed the presence of two main genetic populations along the northeastern Pacific Ocean (northern region with the Hecate Strait and southern region along the west coast of Vancouver Island), with “significant, albeit weak, substructure within regions” (*F*_ST_ = 0.002, *p* = 0.001) (Xuereb et al., 2018). In Bernatchez et al. (2019), six genetic clusters of the eastern oyster (*Crassostrea virginica*) were identified along the Atlantic coast of Canada with significant neutral genetic differentiation (mean *F*_ST_ = 0.0090, *p* < 0.0001) between sampling locations. When assessing diversity across the whole range for the black‐lip pearl oyster (*Pinctada margaritifera*), high levels of differentiation were found between the Indian Ocean and Pacific Ocean basins (*F*_ST_ > 0.25) and within each basin (*F*_ST_ = 0.2534–0.4177, *p* < 0.001, and *F*_ST_ = 0.0007–0.1090, *p* < 0.001, respectively) (Lal et al., 2017). The hierarchical structuring delineated by many of these studies is comparable to the patterns identified here with the hard clam, with a major division between Canadian and US regions and lower levels of divergence between populations within the USA. The levels of differentiation in the current study (mean *F*_ST_ = 0.011, *p* = 0.001) were similar to those found in the Eastern oyster, higher than those found in the American lobster or giant Californian sea cucumber, and much lower than those found with the black‐lip pearl oyster (Benestan et al., 2015; Bernatchez et al., 2019; Lal et al., 2017; Xuereb et al., 2018).

Genetic diversity estimates among the six hard clam populations along the coast of North America were similar. Expected heterozygosity was consistently higher than observed heterozygosity in all groups and both values were relatively uniform across the populations, with positive inbreeding values in all locations (*G*_IS_ 0.087–0.151). The pattern of increased homozygosity and positive inbreeding values that appear as observed heterozygote deficiencies are common in marine bivalves (Bernatchez et al., 2019; Lal et al., 2017; Mallet et al., 1985; Reece et al., 2004). In highly fecund marine species, heterozygote deficiencies are often attributed to a large variance in reproductive success among individuals from different age classes, resulting in population substructure among age classes (Bernatchez et al., 2019; Hedgecock & Pudovkin, 2011). This is a possible contributor to the heterozygote deficiency and positive inbreeding observed in this study that cannot be addressed due to the lack of age data for individual clams. This pattern of higher expected than observed heterozygosity was also seen in wild hard clams in Florida using seven microsatellite markers (Hargrove et al., 2015). Although the current study found positive inbreeding values in all locations, inbreeding levels were generally lower than what has been reported in other bivalve studies using SNPs (e.g., *Crassostrea virginica*: *F*_IS_ > 0.188; Bernatchez et al., 2019, and *Pinctada margaritifera*: *F*_IS_ > 0.53; Lal et al., 2017). Florida wild hard clams also had higher inbreeding values (*F*_IS_: 0.146–0.321; Hargrove et al., 2015) than those calculated in this study, with the exception of the Massachusetts population (*G*_IS_ 0.151), and while it could be an artifact of marker type used for the analyses, it may also reflect a true difference from a region not sampled in this study.

Although there is no available hard clam census size data to compare to the estimated effective population sizes (*N*_e_), inferences about the effect of genetic drift among the populations can be made by comparing the *N*_e_ values of the clam populations in this study. The populations with the lowest estimated *N*_e_ were Maine and Canada, while the populations in the Carolinas had the highest *N*_e_, suggesting that the effective population size of hard clams decreases with increasing latitude. This pattern of *N*_e_ supports the hypothesis of a northward range expansion of the hard clam from common Pleistocene refugia around the Carolinas (Baker et al., 2008).

## 5 CONCLUSION

This study was the first to use GBS to assess the population structure and genetic diversity of hard clams along a large portion of their native range. Six genetically distinct populations of hard clams were identified: Canada, Maine, Massachusetts, Mid‐Atlantic, Chesapeake Bay, and the Carolinas. Results support the findings of previous genetic studies, which not only identified breaks at Cape Cod and Cape Hatteras (Baker et al., 2008; Dillon & Manzi, 1992) but also identified additional barriers to gene flow between Canada and Maine, and between Maine and Massachusetts. This study also documented fine‐scale geographic differentiation between bayside and oceanside Virginia populations, as well as a pattern of isolation by distance in the Mid‐Atlantic region. With the identification of 4960 novel SNPs markers across all sampling areas, this study creates a genetic baseline for monitoring natural hard clam populations along a large portion of their native range.

These markers also provide a baseline for future research on this economically important species to support fisheries and aquaculture. Highly informative marker subsets can be identified for assay panels that can be used for many applications including stock assignment of individuals from unknown populations. These panels also could be used with aquacultured clams to assess heterogeneity and genetic ancestry, track parentage, monitor genetic diversity, and identify desirable brood‐stock source populations. In addition, how genotypes and genetic populations correlate with environmental data can be examined. Physiological experiments paired with transcriptomic analysis could provide a better understanding of differences in salinity, hypoxia, and temperature tolerances among hard clam genetic populations. Identification of genes associated with traits of interest for selective breeding will benefit the aquaculture industry.

## CONFLICT OF INTEREST STATEMENT

The authors have no conflict of interest to declare.

## Supporting information


Appendix S1.

## Data Availability

The data that support the findings of this study are openly available in the Dryad Digital Repository at https://datadryad.org, reference number DOI:10.5061/dryad.kprr4xh9f.
