# Supplementary material for: Fine‐scale population structure of the northern hard clam (Mercenaria mercenaria) revealed by genome‐wide SNP markers
Source: Evol Appl. 2023 Jul 10;16(8):1422–37. doi: 10.1111/eva.13577 (PMC10445094; doi:10.1111/eva.13577)
Supplement: Supplementary file 1 — Appendix S1. [file EVA-16-1422-s001.docx]

**Supplementary Tables & Figures**

**Supplementary Table 1**. Order of filters for SNP dataset received from Diversity Arrays Technology (DArT Pty Ltd, Canberra, Australia) including threshold for removal and the number of SNPs remaining in dataset post-filtering step. Started with 452 individuals and 153,842 SNPs.

| Filter | Threshold for removal | Number of SNPs post-filter |
| --- | --- | --- |
| Coverage Depth | <5x and >75x | 129,562 |
| Repeatability | <99% | 113,970 |
| Monomorphic SNPs |  | 104,540 |
| Call Rate (Loci) | <98% | 14,413 |
| Secondary SNPs | At random | 8,550 |
| Hamming Distance | <20% | 8,092 |
| Minor Allele Frequency | <1% | 4,994 |
| Call Rate (Individual) | <95% | 449 individuals |
| Dropped Duplicate Sample & Recalculated Locus Metadata |  | 448 individuals |
| Hardy Weinberg Equilibrium | Out in at least 4 locations at *p*-value = 0.01 | 4960 SNPs |
| Individual Heterozygosity |  | None removed |
| END |  | 4960 SNPs; 448 samples |

**Supplementary Table 2.** Results for the 18 loci that had matches for sequences producing significant alignments from the Megablast query; outlier locus name, accession number and version, percent identity, alignment length, E-value, Bit Score, and description of the accession number.

**Supplementary Table 3.** Pairwise F_ST_ values between sampling locations based on Outlier dataset (lower matrix) and *p*- values (upper matrix) generated by 10,000 iterations. F_ST_ values that are significant are bolded. If significance is different than the full dataset F_ST_ with the same pair of locations, then the *p*-values and F_ST_ value is italicized.
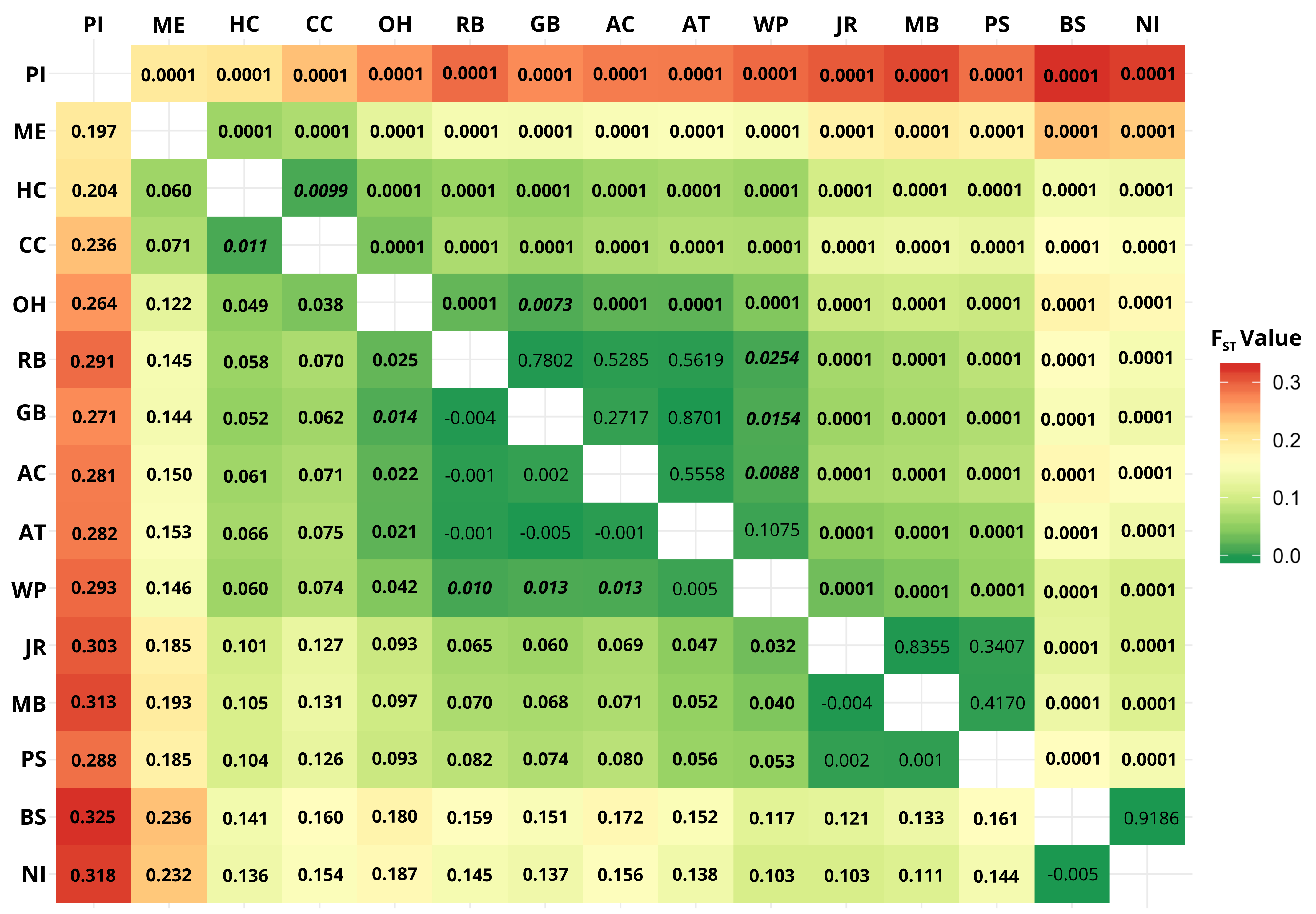


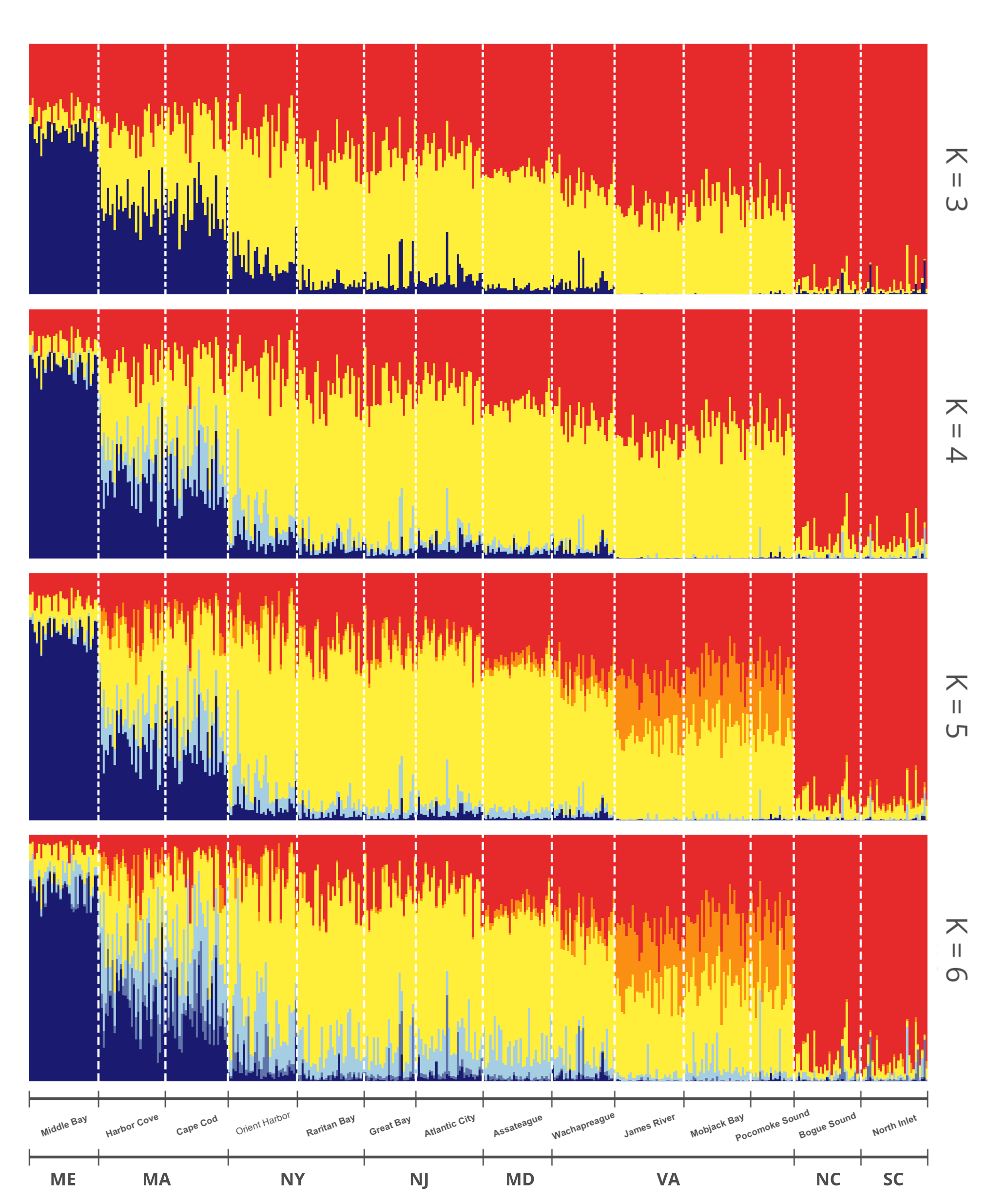


**Supplementary Figure 1**. Structure plot without PI, K = 3 – 6. Each K was run with 500,000 burn-in, followed by 500,000 iterations and 10 replicates each. The admixture model was used, with sampling locations as a prior, and allele frequencies correlated.


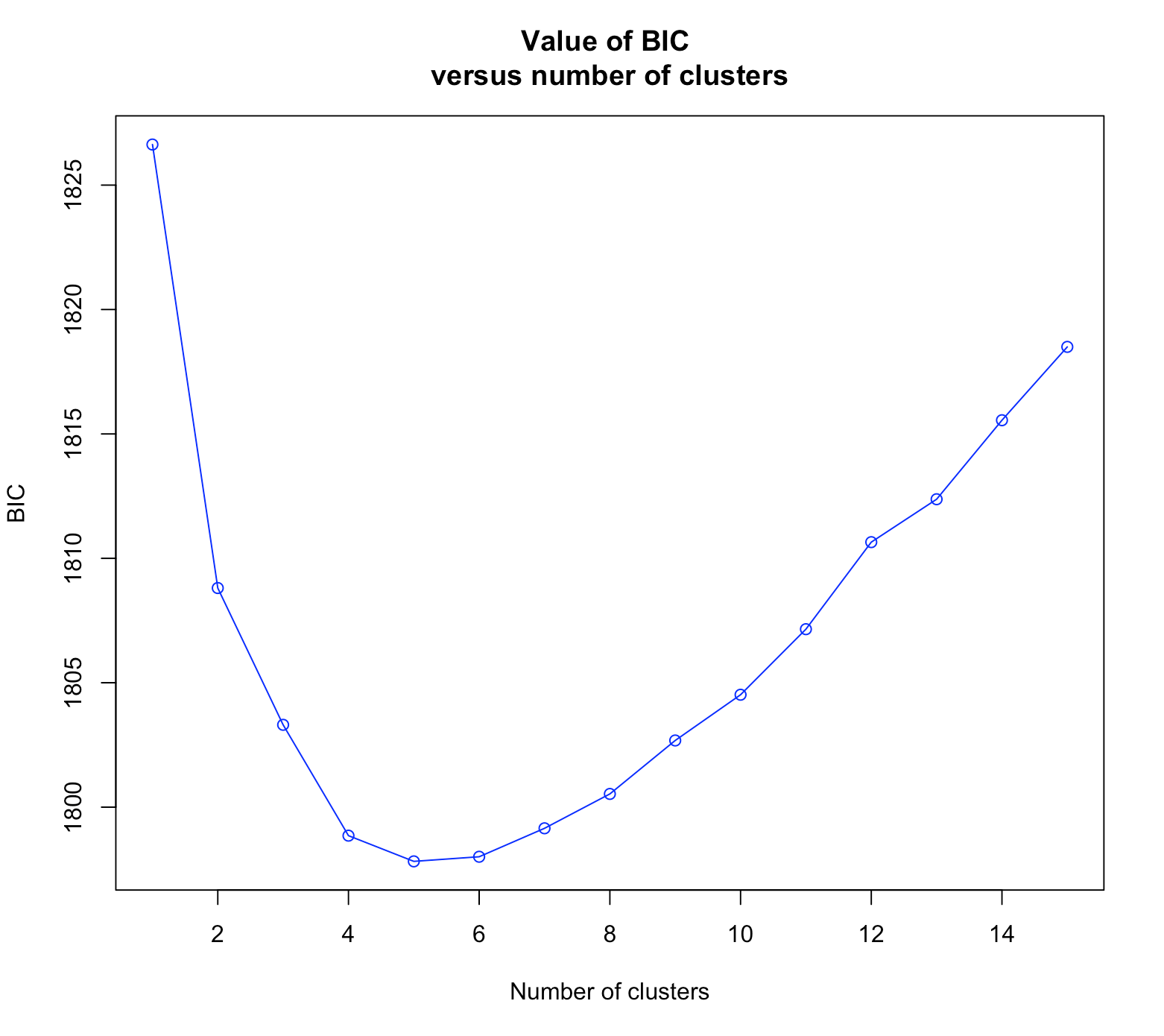


**Supplementary Figure 2.** BIC vs Number of Clusters for Total dataset of all sampling locations: *find.cluster* Analysis for *de novo* optimal cluster identification, K = 5 BIC was most optimal.


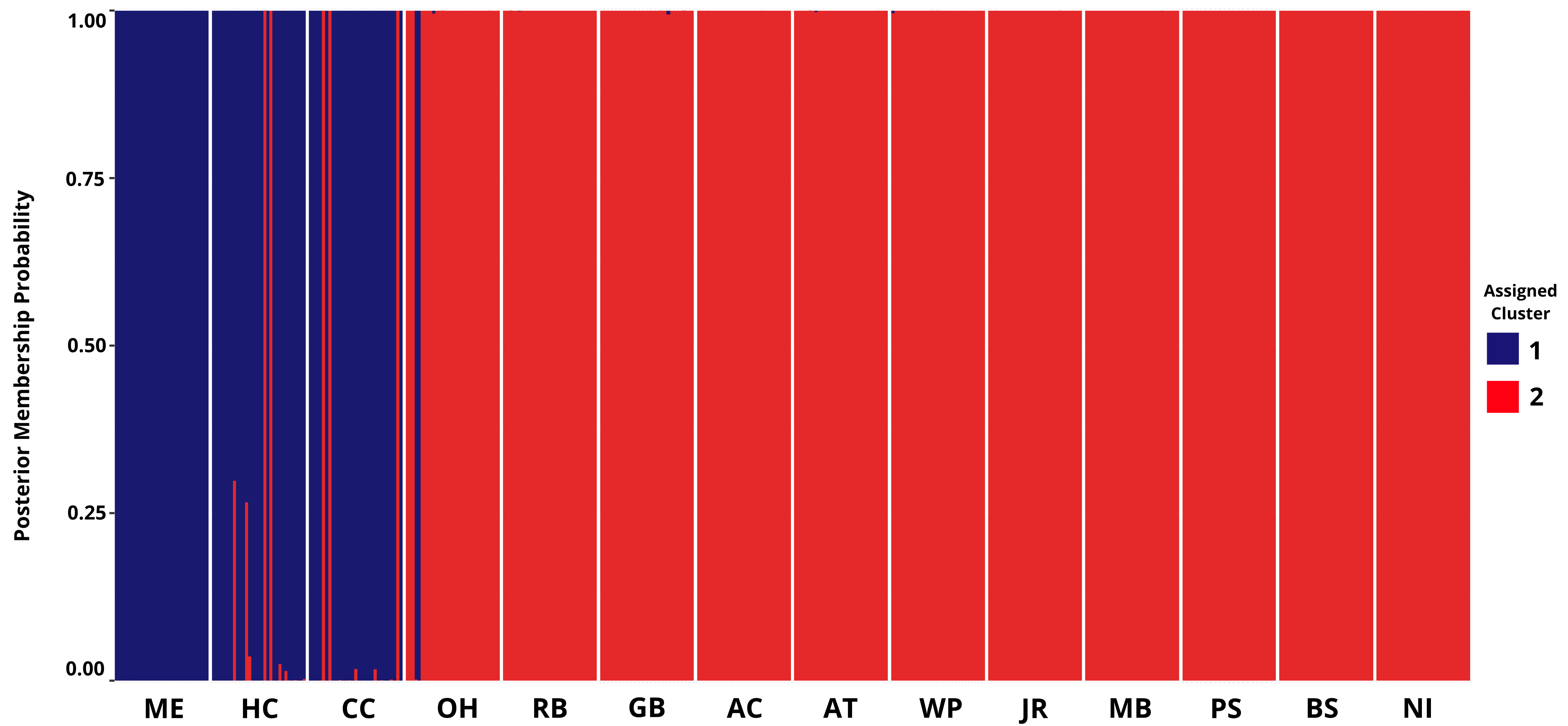


**Supplementary Figure 3**. DAPC of total dataset, no PI, with the most optimal clustering based on BIC, K = 2

**
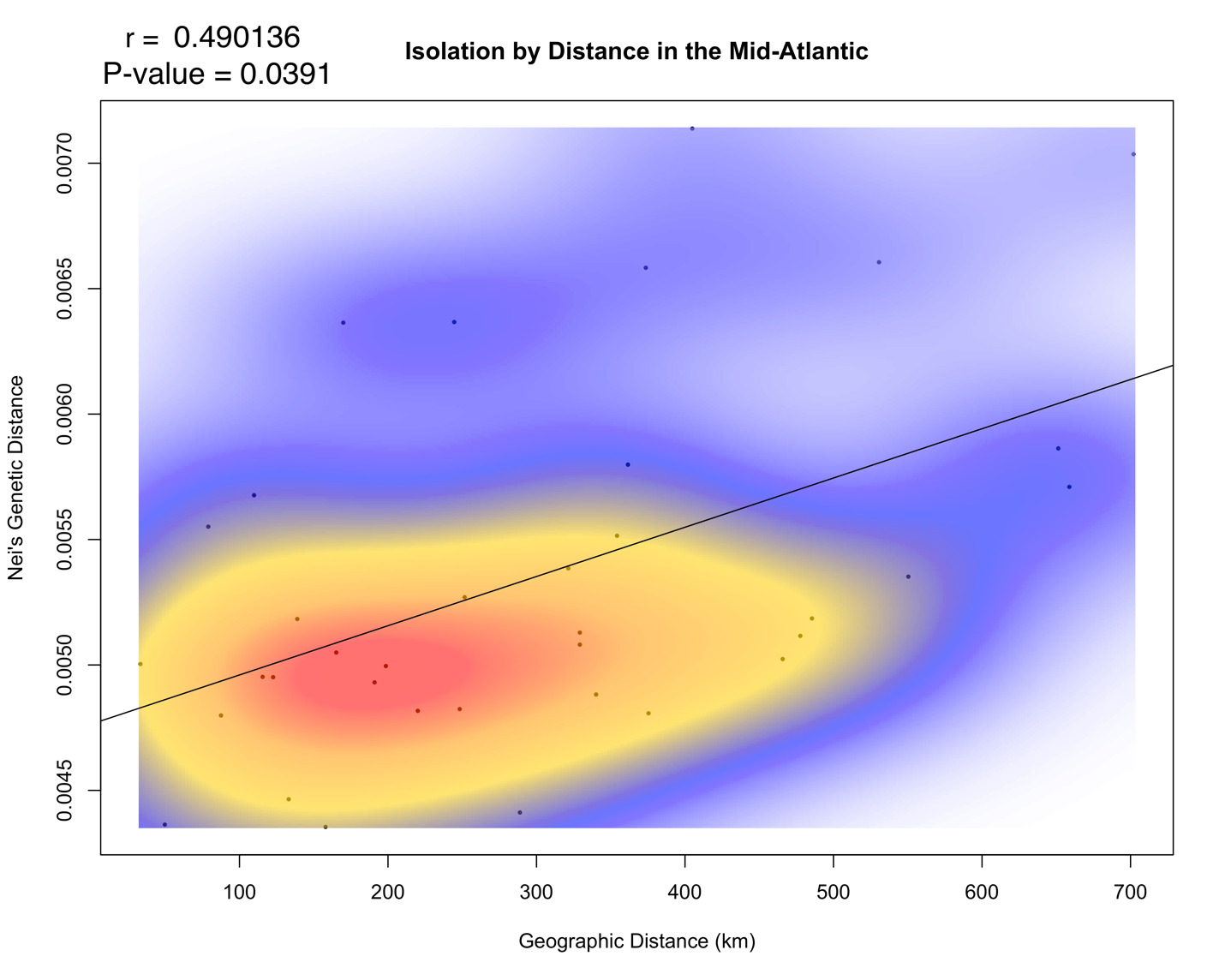
Supplementary Figure 4.** Isolation by Distance in the sampling locations from Orient Harbor, NY (OH) to James River, VA (JR). Shortest over-water distance (km) plotted against Nei’s (1987) pairwise genetic distance.


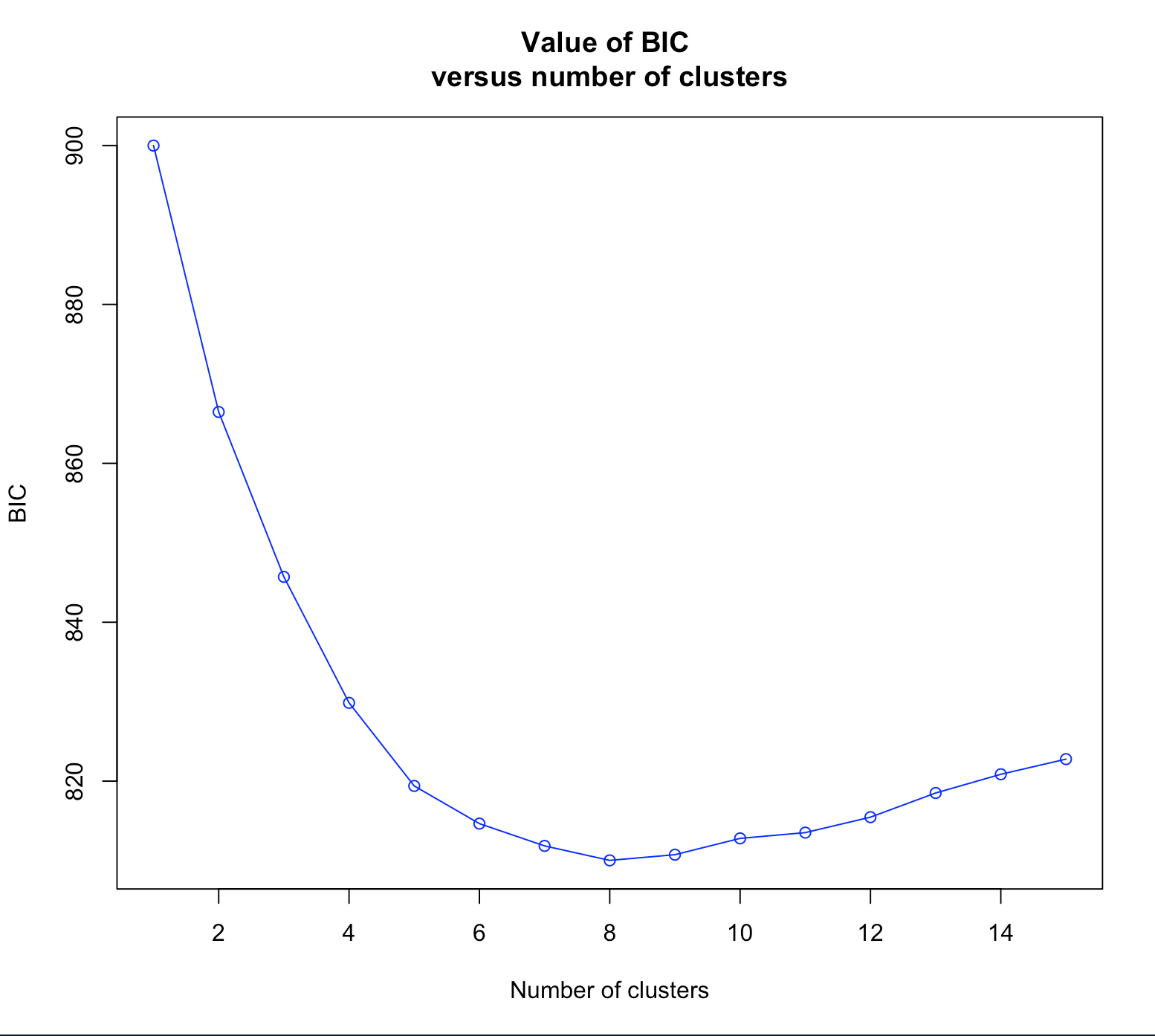


**Supplementary Figure 5.** BIC vs Number of Clusters for Outlier dataset: *find.cluster* Analysis for *de novo* optimal cluster identification, K = 8 BIC was most optimal.
